# Forest genetic monitoring: an overview of concepts and definitions

**DOI:** 10.1007/s10661-016-5489-7

**Published:** 2016-07-29

**Authors:** Barbara Fussi, Marjana Westergren, Filippos Aravanopoulos, Roland Baier, Darius Kavaliauskas, Domen Finzgar, Paraskevi Alizoti, Gregor Bozic, Evangelia Avramidou, Monika Konnert, Hojka Kraigher

**Affiliations:** 1 Bavarian Office for Forest Seeding and Planting, Forstamtsplatz 1, 83317 Teisendorf, Germany; 2 Slovenian Forestry Institute, Vecna pot 2, 1000 Ljubljana, Slovenia; 3 Aristotle University of Thessaloniki, University Campus, 541 24 Thessaloniki, Greece

**Keywords:** Indicators, Verifiers, Genetic diversity, Management, Forest genetic resources, FGM

## Abstract

Safeguarding sustainability of forest ecosystems with their habitat variability and all their functions is of highest priority. Therefore, the long-term adaptability of forest ecosystems to a changing environment must be secured, e.g., through sustainable forest management. High adaptability is based on biological variation starting at the genetic level. Thus, the ultimate goal of the Convention on Biological Diversity (CBD) to halt the ongoing erosion of biological variation is of utmost importance for forest ecosystem functioning and sustainability. Monitoring of biological diversity over time is needed to detect changes that threaten these biological resources. Genetic variation, as an integral part of biological diversity, needs special attention, and its monitoring can ensure its effective conservation. We compare forest genetic monitoring to other biodiversity monitoring concepts. Forest genetic monitoring (FGM) enables early detection of potentially harmful changes of forest adaptability before these appear at higher biodiversity levels (e.g., species or ecosystem diversity) and can improve the sustainability of applied forest management practices and direct further research. Theoretical genetic monitoring concepts developed up to now need to be evaluated before being implemented on a national and international scale. This article provides an overview of FGM concepts and definitions, discusses their advantages and disadvantages, and provides a flow chart of the steps needed for the optimization and implementation of FGM. FGM is an important module of biodiversity monitoring, and we define an effective FGM scheme as consisting of an assessment of a forest population’s capacity to survive, reproduce, and persist under rapid environmental changes on a long-term scale.

## Introduction

The worldwide trend of biodiversity loss at the global, regional, national, and local levels (Graudal et al. 2014; Monastersky 2014) is a reality. As early as in 2002, the European Union formalized the need to act toward halting biodiversity loss and reached specific decisions that are described and embodied in the Decision of the European Parliament and of the Council No. 1600/2002/EC (22 July 2002) laying down the Sixth Community Environment Action Programme. In Article 6 of this Decision, regarding the objectives and priority areas for action on nature and biodiversity, the following objective is foreseen: “halting biodiversity decline with the aim to reach this objective by 2010, including prevention and mitigation of impacts of invasive alien species and genotypes” (EC Official Journal 2002). The challenge seemed to be quite ambitious and hard to meet at the onset, so the succeeding Conference of Parties to the Convention on Biological Diversity (CBD) proposed not to halt but at least to reduce biodiversity loss in a decision stating: “Parties commit themselves to a more effective and coherent implementation of the three objectives of the Convention, to achieve by 2010 a significant reduction of the current rate of biodiversity loss at the global, regional and national level as a contribution to poverty alleviation and to the benefit of all life on earth” (SCBD 2006). By the end of 2010, though, none of the above objectives and milestones had been reached. Yet, high genetic diversity among and within species ensures that forests can grow, adapt, and evolve under environmental changes, but they may face risks in case that changes occur more rapidly than the species’ adaptive and evolutionary mechanisms can absorb. Furthermore, species or population survival is strongly linked to the genetic system. Changes within the genetic system will consequently be manifested at the species and ecosystem levels. The question is how these changes can be detected in a timely manner.

Forest conservation, sustainable use of forest resources, and sustainable management of the numerous forest functions in natural and semi-natural or planted forests are the main goals of monitoring programs in forest ecosystems at the national and international levels. Indeed, European-wide forest ecosystem monitoring started 30 years ago, based on the Convention on Long-range Transboundary Air Pollution (LRTAP, binding from 1983 onward). Later in 1985, the United Nations Economic Commission for Europe (UNECE-CLRTAP) launched the International Co-operative Programme on Assessment and Monitoring of Air Pollution Effects on Forests (ICP Forests 1985). ICP Forests cooperates closely with the European Commission (Regulation 2152/2003-*Forest Focus*), and in the framework of this cooperation, during the last decade, joint projects and developments have been initiated that contribute to monitoring of some aspects of forest biodiversity (Bredemeier et al. 2007; Vilhar et al. 2015).

However, genetic diversity, being a necessary element in the maintenance of biodiversity at all other levels (i.e., species, ecosystem, landscape), has been neglected in all forest monitoring programs to date, mainly because genetic diversity is difficult to measure directly (Schwartz et al. 2006; Graudal et al. 2014). In spite of (i) the CBD (Article 7) that calls for action to “monitor through sampling and other techniques the components of biological diversity” (CBD 1992), (ii) the advancement of the concept of biological conservation to ensure the diversity of species including genetic variation (Allaby 2006), and (iii) an ongoing international scientific debate in forums such as the European Forest Genetic Resources Programme (EUFORGEN, http://www.euforgen.org/), there has been a lack, at least at the natural population level, of approaches regarding the application of genetic indicators in national and international conservation policies (Laikre 2010; Geburek et al. 2010; Aravanopoulos 2011; Graudal et al. 2014). The latest FAO report (FAO 2014) called explicitly for the establishment of monitoring systems in order to improve the information on forest genetic resources. In addition, certification schemes (Forest Stewardship Council (FSC), Programme for the Endorsement of Forest Certification (PEFC)) mention the maintenance of genetic diversity, but no information is provided on how it should be measured and conserved. In order to establish a pan-European network for the conservation of forest genetic resources, EUFORGEN facilitated the development of minimum requirements and data standards for designation of genetic conservation units of forest trees across Europe (Koskela et al. 2013). Later on, the EUFGIS Portal (European Information System on Forest Genetic Resources) was launched, where, on a national basis, forest stands fulfilling the minimum requirements can be designated as dynamic gene conservation units (GCUs) (Koskela et al. 2013) and be inserted in the portal by the EUFGIS national focal points. The GCUs designated following the above framework could be considered as main units on which forest genetic monitoring could be applied and provenance trial indications can be used to identify additional units for FGM (Aravanopoulos et al. 2015). Still, genetic monitoring, within which genetic parameters should be used routinely as indicators, is an essential prerequisite for the maintenance and control of sustainable forest management aiming at the conservation of biological diversity at different levels: within species, species, ecosystem, and landscape.

European forests cover around 215 million ha and provide numerous products and services (Forest Europe 2015). They are very diverse with regard to their biological characteristics, structure, management, and uses. Today, though, forest genetic resources face an array of growing threats. According to the Forest Europe Report on the State of Europe’s Forests, the overall forested area remains rather stable although the variety of threats is increasing (Forest Europe 2015). Climate change, air pollution, unsustainable forest management, invasive species, urbanization, and forest fragmentation are expected to reduce forest biodiversity and may adversely affect genetic diversity and threaten the future adaptive potential and sustainability of European forests and their ecosystems (Rajendra et al. 2014). The authors of the report on the state of Europe’s forests pointed out that “genetic variation in regionally adapted forests is essential for adapting to new environmental conditions such as climate change” (Forest Europe 2015: State of Europe’s Forests), and apart from migration and short-term responses due to phenotypic plasticity, evolutionary responses are required for tree populations to be able to track climate change (Aitken et al. 2008). The presence of strong quantitative genetic research and the growing genomic resources render forest tree species useful for climate change research, despite their long generation times. This is especially true for marginal populations that are facing the largest challenges for adaptive evolution, which are imposed by different factors at the northern and southern edges of the species distributions and may alter the levels and patterns of genetic variation (Florian et al. 2013; Fady et al. 2016). The task for FGM can thus also be to gain better data for developing and assessing models to evaluate accurately whether present genetic variation may enable the populations to respond sufficiently to predicted new climates. The advantage of FGM over other biodiversity monitoring systems is that it can serve as an early warning mechanism for changes that, on higher levels, (species/ecosystem levels) could only be seen later on. It helps to track the temporal and spatial changes of genetic variation of tree populations and shows whether genetic variation can be maintained in the long term and how it can be shaped by overarching dynamic phenomena, such as climate change (Aravanopoulos et al. 2015). Hence, FGM can contribute to the dynamic conservation of genetic diversity over time, by evaluating how the evolutionary processes may affect the adaptive potential of forest tree populations in the case of a change of environmental conditions. However, genetic monitoring is a time-consuming, expensive endeavor, and consequently, the decision on the species to be monitored and the level of monitoring is of great importance. According to Aravanopoulos (2011), Brown et al. (1997), and Laikre et al. (2008), the species selected for monitoring include, among other categories, economically important species, ecologically keystone ones, rare or endangered, and species already included in other monitoring programs. Many forest tree species fall into the abovementioned categories, and for this reason, a substantial body of genetic data and information for forest tree species/populations has been gathered so far (e.g., Petit et al. 2003; González-Martínez et al. 2006; Neale and Kremer 2011), putting foresters in a favorable position that allows them to test and improve the already developed concepts of FGM (Namkoong et al. 1996, 2002; Schwartz et al. 2006; Konnert et al. 2011; Aravanopoulos et al. 2015). However, although some genetic information is available for European forest tree populations regarding genetic variation, gene flow, and hybridization, most studies have not included the temporal aspect of genetic monitoring, i.e., performance of repeated genetic analysis on the same population and in certain time intervals. Therefore, experience on evaluation of the potential changes between assessments is largely missing, although some ideas have been postulated: comparison to reference stands (Namkoong et al. 2002), interpretation of time series of genetic data (Aravanopoulos 2011), and comparison of different generations within stands (Konnert et al. 2011).

Thus, the objective of FGM (i.e., does a specific forest tree population maintain its adaptability to the changing environment in the long run?) may be fulfilled by developing a systematic way to answer questions such as the following: (i) Are the levels of genetic diversity maintained within critical limits? (ii) Will the population be able to survive and reproduce over future generations? (iii) Are there significant changes in the mating system over years? (iv) Does directional change of genotypic frequencies occur over generations? (v) Is the effective population size changing over time? (vi) Are there changes in fructification intensity over the years?

To accomplish the above goals, FGM should include, as a minimum requirement, systematic field measurements of growth traits of old stands and natural regeneration, assessment of the number of reproductively mature trees and seed quality, and recording of phenotypic traits crucial for reproduction and growth, as, for example, flowering onset, termination and intensity, bud break, and bud set. According to Koskela et al. (2013), monitoring efforts should also, ideally, be able to track temporal changes in the genetic variation and structure of tree populations, as this is the only way to verify directly if and how well genetic diversity is maintained over time.

## Forest genetic monitoring in the context of biodiversity monitoring concepts

Biodiversity has been mostly discussed to date at the species and ecosystem level. Genetic diversity within species often is not incorporated in such attempts, although it is the basis of diversity at the species level. Intraspecific genetic variation provides the basis for long-term evolutionary adaptation of populations, mainly through selection mechanisms, and has been assessed by studying adaptive traits (including life history traits) as well as by genetic studies. For example, networks of field trials have been established worldwide for the assessment and improvement of certain economically important traits (e.g., growth, timber uniformity, phenology, essential oil contents, tolerance to biotic or abiotic factors, etc.) (FAO 2014). Results of provenance trials can provide a range for the variation observed for quantitative traits (frost hardiness, bud burst, bud set) in a species’ gene pool across variable environmental conditions. It is important to link molecular and quantitative knowledge in order to understand adaptive traits and, thereby, a species’ adaptive potential. Increasing amounts of data on intraspecific genetic diversity for many tree species are being collected using isoenzyme or molecular markers. Such assessments are needed for conservation, breeding, and use of forest tree species, as genetic polymorphism of adaptive traits is necessary for their survival, by providing the basis for genetic adaptation and selection of desired traits. In genetic monitoring, this might be assessed by using SNPs at outlier loci or, e.g., by reproductive fitness, another selection indicator, to succeed insight into adaptive processes. These observations have not been performed systematically over time, but through comparison of similar developmental stages across populations or different generations within populations (seed, seedlings, adults) (Konnert 1991; Konnert and Ruetz 2006; Bilela et al. 2012; de Morais et al. 2015). Genetic monitoring refers to the systematic observation of genetic processes within a population on a long-term scale and allows us to draw conclusions about gene flow, flowering phenology, etc. over time.

Several concepts of genetic monitoring in forests have been proposed since 1996. Namkoong et al. (1996) proposed an elaborate system that was geared to assist local foresters in sustainably managing forests in terms of genetic diversity. The main idea was to track evolutionary processes in tree populations, which also remained in all the subsequent FGM concepts. Later on, Namkoong et al. (2002) amended the forest monitoring system by adding a number of parameters for measurement and critical reference values for these parameters that could be considered as a signal for undertaking tailored silvicultural and forest management actions. However, the proposed monitoring system was too time-consuming, expensive, and difficult to implement practically in forests, and for these reasons, a simplified but concrete list of parameters was needed, together with guidelines for their interpretation. This was the starting point for further proposals on the FGM concept (e.g., Konnert et al. 2011; Aravanopoulos 2011).

The work of Namkoong et al. (1996, 2002) provided the theoretical basis for the so-called “Concept of a Genetic Monitoring for Forest Tree Species in the Federal Republic of Germany” (BLAG—expert group “Genetic monitoring” 2004) formulated by a working group of forest geneticists, as an essential step for implementing the CBD that called in paragraph 7b for the surveillance of the distinct constituents of biological diversity. In that concept, the state of the genetic system of forest tree populations was proposed to be evaluated by following closely the proposal of Namkoong et al. (1996) and was tested in a pilot study for two model species: European beech (*Fagus sylvatica*) and wild cherry (*Prunus avium*), as reported by Konnert et al. (2011). The German concept is the only one tested in practice so far and provides invaluable outputs for further implementation of FGM on a wider geographic scale and for other tree species. The comparison of recordings of the state of subsequent generations: adult trees, natural regeneration, and seed provided a first sign of the current state of the genetic system of the two studied forest tree species. However, the authors could not agree on whether the examined indicators deserved equal weighting or not. The need to continue with the development and testing of the FGM concept at the national and international levels was suggested (Konnert et al. 2011). Within the European project “FORGER” (completed in 2015), one work package was dedicated partly to the development of FGM measures (http://www.fp7-forger.eu/). The monitoring protocols are still in the testing phase and will be further evaluated and compared with alternative approaches.

During the same year that the first results of the pilot study following the German Concept of a Genetic Monitoring for Forest Tree Species were published, Aravanopoulos (2011) presented a more simplified genetic monitoring system, based partially on the concept of Namkoong et al. (1996), by reducing the number of parameters to be measured. The proposed scheme was a theoretical development based on the “gene-ecological approach,” which showed that most of the important genetic information needed for a comprehensive FGM system could be derived by a considerably reduced number of indicators. EUFORGEN, and in particular the Working Group on Genetic Monitoring, also developed a simplified concept of genetic monitoring based on the Namkoong et al. (1996) concept and the theoretical development of Aravanopoulos (2011) in order to monitor the genetic processes in the European Gene Conservation Units (Aravanopoulos et al. 2015). This concept points out that three options for the assessment of genetic processes should be available, from the most basic and inexpensive (but still indicative) to the most comprehensive, complete and expensive option, utilizing the most recent available molecular methods (Aravanopoulos et al. 2015).

A single aggregated measure to monitor biodiversity has been proposed by Geburek et al. (2010), called the “Austrian Forest Biodiversity Index” (AFBI). The AFBI takes into account different parameters, based mainly on the Austrian forest inventory with different weighting. The weighting is superimposed according to the significance for the maintenance of forest species richness and genetic diversity, but profound and well-documented reasoning for the different weights of each parameter is missing. Some of the presented parameters focus on the maintenance and conservation of genetic diversity (e.g., presence of natural regeneration, forest genetic reserves, seed orchards for vulnerable, rare or endangered species, etc.). One genetic parameter has been included (i.e., mitochondrial marker in *Picea abies*) to predict the most likely autochthonous plant material for a certain Austrian region, and Geburek et al. (2010) proposed that, in the future, improved molecular tools should be used.

However, the present paper is not devoted to giving a comprehensive overview of biodiversity indices (e.g., Scholes and Biggs 2005) but rather to discussing genetic monitoring as a tool to assess the basic component of biodiversity. In the future, several concepts and monitoring approaches should be combined and evaluated in an integrated manner, but our attempt is to establish and implement a FGM system as a starting point.

## Definitions, criteria, indicators, and verifiers

### Definitions

Genetic monitoring, according to Schwartz et al. (2006), refers to “quantifying temporal changes in population genetic metrics or other population data generated using molecular markers.” The definition used by Schwartz et al. (2006) divides genetic monitoring into two categories. The first category includes monitoring based on diagnostic molecular markers for traditional population monitoring (identification of individuals, species, and populations), while the second category refers to monitoring of population genetic parameters. Laikre et al. (2008) required conservation genetic monitoring to include “systematic, temporal study of genetic variation within particular species/populations with the aim to detect changes that indicate compromise or loss of genetic diversity.” They mainly focused on species being subjected to large-scale exploitation, involving breeding-release and/or harvest (removal or addition of individuals with particular genotypes and phenotypes), that are known to have profound effects on the genetic characteristics of populations. Aravanopoulos (2011) defined genetic monitoring as the “quantification of temporal changes in population genetic variation and structure, generated by measurement of appropriate parameters.” Genetic monitoring according to Konnert et al. (2011) is the “observation of the dynamics of transition from the present to the future genetic status.” Following the abovementioned definitions, genetic monitoring, more specifically FGM, entails the spatial and temporal recording of the state of the forest genetic resources and the interpretation of any changes observed. Therefore, only a single temporal insight into the structure and genetic variability of a population cannot be and is not genetic monitoring. However, in forests, at least two generations can be found at the same place and time. In uneven aged continuous cover forests, more overlapping generations are often present due to different reproductive years contributing to the natural regeneration. Furthermore, natural regeneration can be influenced by annual variation in flowering abundance as well as flowering synchronization, which typically have a genetic and an environmental component, affecting the genetic composition of the seed crop and the subsequent natural regeneration in a forest stand (Nikkanen and Ruotsalainen 2000; Konnert and Behm 1999; Alizoti et al. 2010). These generations pass through different individual tree developmental phases (i.e., seeds, seedlings, saplings, pole trees, adults—reproducing trees) and also form different forest stand developmental phases (i.e., stands in rejuvenation, establishment stage, pre-thicket, thicket, pole, and timber stage (SILVATERM 2015), which can serve as a proxy to understand the evolving processes governing genetic variability and structure of a certain forest stand.

### Criteria, indicators, and verifiers

Monitoring programs need well-defined objectives. An objective is usually reflected in a criterion. A criterion is a comprehensive objective that can be judged, but it is not a direct measure itself. Namkoong et al. (2002) proposed a single criterion for forest genetic monitoring: “conservation of the processes that maintain genetic variation.” An indicator is any forest ecosystem component or process that can describe the trends of sustainability of the resource and be used as a sign to assess whether the associated criteria are being met (e.g., directional change in allele frequencies, maintenance of the mating system, etc.) (Boyle 2000). An indicator can be measured periodically to capture the potential changes within the ecosystem. Finally, a verifier is a measure of a parameter that adds meaning and precision to the indicator, providing a quantitative target, objective, or threshold that needs to be met (e.g., number of alleles, level of inbreeding, etc.) (Gardner 2010).

So far, no generally accepted indicators have been proposed for practical use on the international level (Graudal et al. 2014). To cover this gap, Graudal et al. (2014) proceeded with the identification of four types of indicators: state, pressure, benefit, and response. State indicators deal with the status and condition of biodiversity and whether it is changing, by analyzing genetic diversity, i.e., where and how genetic diversity is lost. Pressure indicators deal with the causes of biodiversity loss, by monitoring their extent and intensity. Benefit indicators pinpoint the benefits of biodiversity to human societies and the costs of its loss by quantifying them, while response indicators are meant to reflect the reaction of the society, by measuring the implementation of policies or actions to prevent or reduce biodiversity loss. The above authors also suggested specific indicators that are ecological and demographic measures of adaptive diversity (verified by, e.g., age/size class distribution, number of reproducing trees, abundance of regeneration, etc.) and molecular marker assessment (verified by, e.g., effective population size, allelic richness, etc.). Following the Strategic Plan for Biodiversity 2011–2020 (UNEP/CBD/COP 2010, 2011), Graudal et al. (2014) elaborated on meaningful and realistic genetic diversity indicators with different emphasis on the type of question that they are intended to address. State indicators are considered fundamental not only for describing the state of genetic diversity change but also, if sequentially assessed, for the temporal component of genetic diversity change. Indirect indicators of pressure, benefit, and response should then be built upon state indicators to monitor the extent and direction of genetic diversity change, to quantify the benefits of genetic diversity for humans, and to identify the implementation success of policies and actions to prevent loss of genetic diversity. The indicators proposed in current theoretical concepts (Namkoong et al. 2002; Aravanopoulos 2011; BLAG Expert Group 2004) and put into practice for two species in Germany (Konnert et al. 2011) refer to such state indicators, describing demographic and genetic conditions of selected populations. A combination of state (e.g., deadwood) and response (e.g., natural forest reserves, seed stands) indicators to monitor biodiversity has been proposed by Geburek et al. (2010).

The abovementioned concepts of FGM differ in the number of indicators and verifiers. Namkoong et al. (1996) suggested the first forest monitoring system, based on four indicators (drift, selection, mating system, and migration) and 18 verifiers. Later on, Namkoong et al. (2002) amended their monitoring system and included 14 demographic and nine genetic verifiers to describe the four indicators. The German concept uses the four indicators (level of genetic variation, directional change of gene or genotypic frequencies, changes in mating system, and gene migration between populations), proposed by Namkoong et al. (1996) and a number of verifiers; for a pilot study in beech and cherry, 18 and 14 verifiers were used, respectively (Konnert et al. 2011). Aravanopoulos (2011) suggested three indicators (natural selection, genetic drift, and gene flow-mating system) and seven verifiers to measure these indicators. The proposed concept of the EUFORGEN Working Group on Genetic Monitoring includes two indicators (selection; genetic variation-mating system) and a set of 11 verifiers (Aravanopoulos et al. 2015). Finally, Konnert et al. (2011) concluded that the indicators applied in their pilot study can be useful for estimating genetic implications of forest management actions, for developing practical strategies concerning the conservation of forest genetic resources, and for integrating genetic aspects in the strategic utilization of different monitoring programs (Konnert et al. 2011). A detailed overview of proposed indicators and verifiers from the concepts of Konnert et al. (2011), Aravanopoulos (2011), Graudal et al. (2014), and Aravanopoulos et al. 2015 is presented by Aravanopoulos (2016). As an example, the indicator “selection” was suggested to be measured by age and size class distribution, by reproductive fitness (percentage of filled seeds and percentage of germination), and by regeneration abundance in the concept of Aravanopoulos (2011), whereas Konnert et al. (2011) suggested “directional change in gene/genotypic frequencies” to be measured by allele, genotype, phenotype frequencies, and distribution of age classes. The goal of the LIFEGENMON project (LIFE for European Forest GENetic MONitoring System; www.lifegenmon.si) is to condense these sets of indicators and verifiers to the most meaningful and cost-effective ones in a transnational case study approach.

## Perspectives for the design of FGM and its implementation into practice

There is a clear need (e.g., Koskela et al. 2013, FAO 2014) and mandate (through CBD from 1992 onward; CBD 1992) to design and implement a forest genetic monitoring system based on informative and cost-effective indicators and verifiers. Within the LIFEGENMON project, indicators and verifiers fulfilling the above requirements are to be identified and FGM will be implemented on a transnational scale, on a transect extending from south Germany (Bavaria) to Greece. By introducing genetic monitoring into conservation programs and sustainable forest management schemes, the assessment of information related to adaptive and neutral genetic diversity changes over time, on a species and/or on a population basis, is becoming a tangible goal. The indicators and verifiers that will be used for genetic monitoring will also serve as early warning signals for the assessment of the species/population’s response to environmental changes in the long run. The LIFEGENMON project started in 2014 and will last until 2020. The expected outputs of LIFEGENMON are guidelines for the FGM of seven selected tree species, an implementation manual, and a decision support system that will provide guidance on how to implement an optimal forest genetic monitoring solution to all interested stakeholders across different countries and regions. Another goal is the production of background professional documents for the potential development of future legislation and strategies, aiming toward the further development of measures for adaptive forest management in terms of protection and sustainable use of forest genetic resources.

**Fig. 1:**
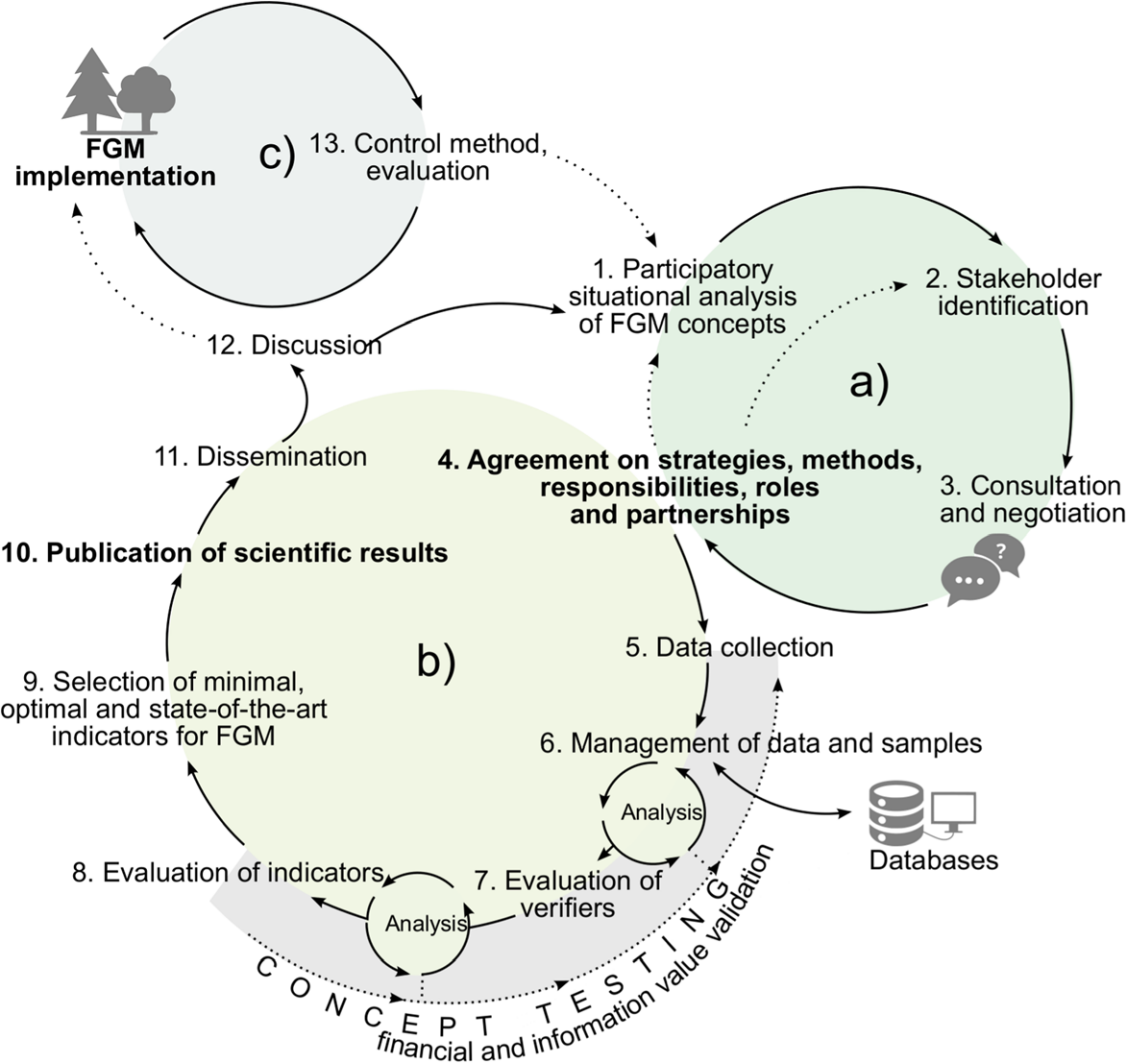
Development and implementation of the FGM concept (Adapted from Finzgar et al. (2015) and printed with permission from the publisher)

The following outlined scheme developed within the LIFEGENMON project (Fig. 1) was designed to lead to the successful establishment and implementation of FGM. According to this scheme, the FGM concept will be developed in two phases. In phase (a), before the implementation of FGM, a review of the current state of existing FGM concepts, expert opinions, practical experience, and draft guidelines on forest genetic resources will be completed. The conclusions achieved during the review process will be discussed among identified stakeholders, and a new FGM concept will be negotiated. With a general agreement on strategies, methods, responsibilities, roles, and partnership between the stakeholders, the working environment becomes better organized and provides better results in phase (b). In phase (b), the agreed-upon FGM concept will be tested by assessing demographic and genetic parameters on selected monitoring plots and by fine-tuning different sampling and laboratory methods. By following this procedure, collection of a large amount of data is underway and will continue till the finalization of the project; the data is being systematically stored in standardized databases. During this optimization phase, validation of selected methods (both financial and informational ones) takes place. After optimization, a range of minimal, optimal, and state-of-the-art indicators and verifiers can be identified, in order to achieve the objectives stated in a criterion. After the scientific analysis and the results on the tested FGM concept are published, they will be further disseminated to different target groups so that they can be better introduced into the FGM concept. If the results are not the expected ones or the discussion among the target groups generates negative reviews, the concept can return to phase (a) for further amendment; otherwise, it can be implemented as a routine monitoring task. The concept is evaluated after every sampling/testing period (c). Similarly, to the control method (Biolley 1920), the concept is then updated based on the new results. If these results are not meeting the FGM objectives, the concept can be revised and redesigned in phase (a).

## FGM as a tool for conservation and management of forest genetic resources

Knowledge about the genetic impact of forest management systems that might be different in temperate, boreal, and tropical forests is crucial for conservation and management of forest genetic resources (Ratnam et al. 2014). However, for an effective monitoring program with respect to detection of human impact, it is necessary first to assess the baseline data, i.e., random fluctuations of genetic structure of a population, in order to be able later on to detect genetic changes caused by anthropogenic factors (Charlier 2011). Gene flow is a major determinant of the impact of forest tree plantations on the surrounding wild populations and ecosystems. Higher impact on the whole genetic resource, though, is expected when local populations are small, in marginal conditions or in unpredictable environments (Lefevre 2004). Realistic prediction of gene flow is therefore essential for risk assessment of the potential impacts of plantations on natural populations. Methods for measuring gene flow include parentage analysis and analysis of genetic diversity in seed and seedlings, for which data can be obtained through genetic monitoring. Introduced tree species can also be the target of genetic monitoring in order to follow their development (e.g., reproduction and regeneration success) and their potential ecological impact on wild populations through hybridization and introgression.

Over the last few decades, forests have been facing many threats and pressures, especially those related to climate change and overexploitation. To fully understand how these threats affect the sustainable use of forests and their conservation, FGM should be used as a primary tool. Materialization of FGM concepts, in the framework of forest ecosystems research, has been delayed for a long time. If we consider that sustainable forest management is based on long-term adaptability of forest ecosystems, starting at the gene level, the development of fundamental principles for FGM is an important step toward conservation and sustainable use of forest genetic resources and forest ecosystems. Every approach to FGM should include the development of the concept and its implementation through time and/or after silvicultural actions and natural disturbances. In this way, FGM is destined to assist not only pan-European efforts and national gene conservation programs, but also global-scale strategies on development and implementation of forest genetic resource monitoring. In addition, FGM will help forest managers to maintain adaptability through measures that will secure high genetic diversity levels in their forests. The results of FGM can contribute to the optimization of genetic diversity management, in view of the sustainable utilization of forest ecosystems and managed forests (Konnert et al. 2011), and can add to the sustainable use of natural resources. In addition, FGM is an important tool for directing further research on determining the causes of the different patterns of genetic processes observed, as well as for answering specific hypotheses about possible causes of observed population changes.

Human impact on the genetic structure of forest tree populations can vary significantly. For example, artificial regeneration (especially from unknown sources or using forest reproductive material of limited genetic diversity) is one of the most obvious silvicultural practices that can influence the genetic structure of future forests, while the gene flow that will follow from the planted genetic material will have an impact on the genetic structure of neighboring forests (Finkeldey and Ziehe 2004; Konnert and Hosius 2010; Ratnam et al. 2014). However, natural regeneration can also result in changes of genetic structures, as, for example, when population sizes are seriously reduced through severe felling of reproductive trees or when population densities in rare or scattered species are low, leading to less diverse parental combinations in the offspring. Finkeldey and Ziehe (2004) described possible consequences on the genetic structure of subsequent generations due to target diameter felling. Especially in tropical countries, where the detrimental consequences of silvicultural management practices on genetic structures are most severe (Finkeldey and Ziehe 2004), the shrinkage of forest cover and the ongoing forest fragmentation are adding to the loss of genetic resources in the world’s most diverse ecosystems (FAO 2001, 2014; Finkeldey and Ziehe 2004; Ratnam et al. 2014). Ratnam et al. (2014) reviewed the impact of forest management practices on genetic diversity for temperate, boreal, and tropical forests based on experimental and simulation studies. They concluded that the impact on genetic diversity depends on manifold factors such as the management system applied, stand structure, species’ distribution, and demography. The additional value of FGM for forest managers is that the detected changes in population genetic structure can provide feedback for changes in the population management, by re-evaluating and adapting the existing management practices (e.g., Kelleher et al. 2015).

For tropical forests, Finkeldey and Hattemer (2007) emphasized FGM to be an integral part in tracking management and conservation measures and Dawson et al. (2014) focused, in particular, on management of forest genetic resources with respect to the value of trees to rural communities. Dawson et al. (2014) formulated three suggestions based on different production categories: (i) greater understanding of genetic aspects, especially gene flow, is needed, (ii) more attention has to be paid to genetic quality of planted trees, and (iii) more emphasis should be put on the valuation of wild and semi-wild forest genetic resources. They concluded that genetic variation within species has not been properly considered in the management and use of tropical tree species.

Despite the differences in management systems and biological attributes among tropical, temperate, and boreal forests, one genetic monitoring scheme can be applied in all. There might be some particularities among tree species such as spatial distribution (density), mating system (e.g., the ways of pollination in tropical forests), shade tolerance, etc. Some difficulties may also occur during genetic monitoring of tropical forest tree species because of their high number and identification ambiguities (Ratnam et al. 2014). However, the extent, role, and importance of tropical forests are great and some research on the basic requirements for forest genetic monitoring (e.g., species identification, molecular marker development, gene flow) is currently in progress (Kremer et al. 2005; Dick et al. 2008; Quesada et al. 2013; van Zonneveld et al. 2014; Russell et al. 2014). Certainly, the FGM system developed for European forests should be adjustable to the specific requirements of other forest types as well.

Ratnam et al. (2014) provided very detailed recommendations on the genetic methods and measures that should be used to obtain a comprehensive view of the genetic impacts of forest management practices on temperate, boreal, and tropical forests. They recommend development of a field guide for sustainable management of genetic diversity, which would also include monitoring of flowering phenology and synchronization. The above goes hand in hand with current concepts of FGM and their objectives, as well as with the definition presented in this paper, where FGM by the observation of the genetic system entails assessment of a forest population’s capacity to survive and reproduce in a given environment. Genetic diversity is best conserved within natural habitats of wild plants, but in the case of tropical forests, it may be highly threatened due to the current processes of large-scale habitat destruction. In any case, in a recovery program, the genetic aspect should not be ignored. In tropical forests, FGM is a key element for facilitating population recovery providing new methodologies and new management guidelines that can be used for conservation of forest genetic diversity.

European forests that have been strongly affected by numerous anthropogenic influences and are expected to be strongly affected by the ensuing climatic change are in immediate need of a genetic monitoring scheme. Several approaches exist, which have to be harmonized at the European scale. Within the LIFEGENMON project, guidelines will be developed on the basis of different intensity and cost levels and will be prepared for implementation in practice by training courses. However, due to the development of new genetic/genomic tools, contributing to the more detailed information on the genetic structure of forest tree populations, and of new quantification methods and means, FGM should remain open for further improvement.
